# Leading co-production in five UK collaborative research partnerships (2008–2018): responses to four tensions from senior leaders using auto-ethnography

**DOI:** 10.1186/s43058-022-00385-0

**Published:** 2023-01-27

**Authors:** Peter van der Graaf, Roman Kislov, Helen Smith, Joe Langley, Natalie Hamer, Mandy Cheetham, Daniel Wolstenholme, Jo Cooke, Sue Mawson

**Affiliations:** 1 Northumbria University, Newcastle upon Tyne, UK; 2 Manchester Metropolitan University, Manchester, UK; 3 University of Manchester, Manchester, UK; 4 National Institute for Health and Care Research, Liverpool, UK; 5 Sheffield Hallam University, Sheffield, UK; 6 Newcastle University, Newcastle upon Tyne, UK; 7 Royal College of Obstetricians and Gynaecologists, London, UK; 8 Sheffield University, Sheffield, UK

**Keywords:** Co-production, NIHR CLAHRCs, Applied health research, Auto-ethnography

## Abstract

**Background:**

Despite growing enthusiasm for co-production in healthcare services and research, research on co-production practices is lacking. Multiple frameworks, guidelines and principles are available but little empirical research is conducted on ‘how to do’ co-production of research to improve healthcare services. This paper brings together insights from UK-based collaborative research partnerships on leading co-production. Its aim is to inform practical guidance for new partnerships planning to facilitate the co-production of applied health research in the future.

**Methods:**

Using an auto-ethnographic approach, experiential evidence was elicited through collective sense making from recorded conversations between the research team and senior leaders of five UK-based collaborative research partnerships. This approach applies a cultural analysis and interpretation of the leaders’ behaviours, thoughts and experiences of co-production taking place in 2008–2018 and involving academics, health practitioners, policy makers and representatives of third sector organisations.

**Results:**

The findings highlight a variety of practices across CLAHRCs, whereby the intersection between the senior leaders’ vision and local organisational context in which co-production occurs largely determines the nature of co-production process and outcomes. We identified four tensions in doing co-production: (1) idealistic, tokenistic vs realistic narratives, (2) power differences and (lack of) reciprocity, (3) excluding vs including language and communication, (4) individual motivation vs structural issues.

**Conclusions:**

The tensions were productive in helping collaborative research partnerships to tailor co-production practices to their local needs and opportunities. Resulting variation in co-production practices across partnerships can therefore be seen as highly advantageous creative adaptation, which makes us question the utility of seeking a unified ‘gold standard’ of co-production. Strategic leadership is an important starting point for finding context-tailored solutions; however, development of more distributed forms of leadership over time is needed to facilitate co-production practices between partners. Facilitating structures for co-production can enable power sharing and boost capacity and capability building, resulting in more inclusive language and communication and, ultimately, more credible practices of co-production in research. We provide recommendations for creating more realistic narratives around co-production and facilitating power sharing between partners.

**Supplementary Information:**

The online version contains supplementary material available at 10.1186/s43058-022-00385-0.

Contributions to the literature

- Despite an abundance of frameworks and models, there is noticeable gap in the current literature on ‘how-to’ do co-production in large partnership structures
- Our paper identifies four tensions in doing co-production of research which senior leaders need to solve to create a realistic narrative for their partnerships
- The four tensions help collaborative research partnerships to tailor co-production practices to their local needs and existing opportunities
- Variation in co-production practices should not be reduced to one gold standard but celebrated
- More distributed forms of leadership are needed to facilitate power sharing between partners

## Background

Interest in and use of co-production in healthcare services and research is growing. Funders of applied health research have embraced co-production as a means of improving patient, public and professional involvement [1–3]. Academics have been equally enthusiastic in developing a range of conceptual frameworks, guidelines and principles for co-production, underpinned by a rich and growing literature on the topic, with insight from the social sciences and humanities [4], political science [5], public management [6] and academic entrepreneurship [7] literature. Recent systematic reviews of co-production have summarised the different co-production approaches in use and collated the outcomes and effects of co-production [8].

These reviews show a plethora of terms in use; for example, within healthcare we see services, programmes and interventions being ‘co-created’, ‘co-designed’, ‘co-evaluated’ or ‘co-implemented’ and often authors used these terms in combination to describe their work [8]. This can involve stakeholder and public engagement through participation or involvement in any or all steps of the applied research cycle [9, 10]. All are regarded as processes of co-production but the way they are enacted and operationalised varies depending on the purpose, what is being co-produced and by whom [11, 12]. Some of the ambiguity in co-production also comes from its unclear relationship with patient and public involvement/and engagement (PPI/E) [13]. Other structural approaches, such as Experience-Based Co-Design (EBCD), appear to be more often applied to service development, whilst community engaged research dissemination [14] seems to have a more limited focus on dissemination of research findings. In this paper, we are selecting ‘coproduction’ as the umbrella term, acknowledging that this concept is hard to define given the plethora of definitions and approaches in circulation, and by having both instrumental and moral value [15].

Despite the proliferation of conceptual thought, empirical studies on co-production are less frequent [16]. Many of co-production models and frameworks are not supported by robust evidence [17] and do not describe in practical terms what co-production of research on the ground looks like [18]. It is therefore timely to reflect on what has been learnt about the practice of co-production in applied health research and to help shape the direction of future research.

In the UK context, some argue that the architecture of the new NIHR Applied Research Collaboration funding model enables authentic and visible co-production [19]. Others are more cautious, arguing that co-production can only be as successful as the system allows, and that traditional research structures often fail to facilitate effective public involvement, leading to co-opting of the term co-production without making a tangible difference to professional practices and health outcomes for service users [15, 20]. There are anecdotal accounts of successful collaborative working from the previous NIHR funding model, Collaborations for Leadership in Applied Health Research (CLAHRCs), who were evidence-based following the Knowledge to Action model [21] to ensure that all resulting interventions or findings were underpinned by robust research evidence. These accounts suggest that co-production projects added value and led to the implementation of novel services and interventions [22, 23]. This model also introduced a focus on leadership and governance for co-production that we will explore in more detail in our paper. So-called ‘success’ stories like these are not always published or reported on or described in a way that explicates how best to support researchers to co-produce applied health research or complex health interventions [24].

Therefore, this paper brings together insights from those in leadership positions in collaborative research partnerships in the UK on practising co-production with the aim to inform practical guidance for new partnerships facilitating the co-production of applied health research in the future. The focus of this paper is on the co-production of healthcare services, which aims to collaboratively produce and apply knowledge involving academic researchers as well as health practitioners and policy makers in local government (LG) to inform service development and decision making, with the active inclusion of all partners in the research design and process [25]. This approach is indebted to the work of Elinor Ostrom [26], who used the term co-production to describe a process through which ‘inputs from individuals who are not “in” the same organisation are transformed into goods and services’. This approach blurs the boundaries between ‘knowledge production’ and ‘knowledge application: the former often focuses on researchers’ roles, whilst the latter is of most value to health practitioners and policy makers (knowledge translation and problem-solving). Co-production through collaborative research partnerships helps to bring the two approaches together.

## Methods

Using an auto-ethnographic approach [27], experiential evidence was elicited through collective sense making from conversations between the research team and senior leaders of five collaborative research partnerships, including four former CLAHRCs (Yorkshire & Humber, Greater Manchester, East Midlands and South London) and one former UK Clinical Research Centres (UKCRC) research centre of excellence (Fuse, the Centre for Translational Research in Public Health). These collaborative partnerships were selected from a convenience sample through our shared participation in a special interest group on co-production.

### Five collaborative research partnerships

NIHR CLAHRCs were created in 2008. The NIHR initially funded nine CLAHRCs across England with a specific aim: to develop and conduct applied health and care research across the NHS and to translate research findings into improved outcomes for patients [24]. Each individual CLAHRC did this by creating linkages and partnerships between the applied health and care researchers who conduct the research, and those who use the research in practice, developing different practices of co-production. In 2013, following the success of the pilot CLAHRCs, NIHR funded a second round of 13 CLAHRCs for a 5-year period starting in January 2014. CLAHRCs were each structured into thematic programmes (themes) bringing together researchers, practitioners and patients with shared interests through regular meetings and events.

Fuse was established in 2008 as one of five public health research centres of excellence in the UK funded by the UKCRC collaboration. Fuse works across five universities in the North East of England with a prime focus on the production of excellent research, and its translation into usable evidence to inform practice. The Centre applies a 5-step model to knowledge exchange that encourages co-production of research between partners, including a rapid responsive research and evaluation service [28].

### Data collection

Data on the five collaborative research partnerships is drawn from recorded online interviews between the research team and senior leaders of these partnerships between April and July 2021. Theme leads within each former CLAHRC and Fuse with responsibility for co-production of research activities within their region were identified through personal networks of the research team and invited by email for an online interview. Five theme leads agreed to a recorded semi-structured interview, followed by informal email conversations, and gave consent for the interviews to be recorded. In the interviews, we aimed to document the learning from a selection of CLAHRCs and similar partnerships, and to draw up narrative accounts around their experiences, as we wanted to understand the overall leadership narrative around co-production. Interviews followed a story line topic list (Additional file 2: Appendix 1). Participants were not provided with a definition of co-production upfront but were asked in the interviews to reflect on approaches to co-production adopted within their partnerships. Inductive data analysis was used to determine how different partnerships thought of co-production and to compare different descriptions and practices.

### Data analysis

Recorded online conversations were transcribed and analysed using an auto-ethnographic approach [27]. Auto-ethnography is a research method that uses a researcher’s personal experience to describe and critique cultural beliefs, practices and experiences. It acknowledges and values a researcher’s relationships with others and shows ‘people in the process of figuring out what to do, how to live, and the meaning of their struggles’ [29]. Auto-ethnography is a self-reflective form of writing that has been used across various disciplines such as communication studies, sociology, psychology, organisational behaviour, nursing and paramedicine. In this study, we used this approach to apply a cultural analysis and interpretation of the leads’ behaviours, thoughts and experiences of co-production between 2008 and 2018 in relation to the academics, health practitioners, policy makers and local communities/third sector organisations involved in co-produced research projects within the collective research partnerships.

This method was chosen in recognition of the sensitive nature of the dialogues that take place between programme leads and the research team and the importance of these dialogues for collective sense making of co-production practices. The auto-ethnographic approach allowed for a safe deconstruction of these conversations that was sensitive to the research team’s own input to these conversations.

The transcribed data were analysed in three steps: starting with individual recall and reflection by each author, followed by joint analysis with the research team of the transcribed conversations, and finally, collective sense making with the interviewed CLAHRC programme leads in an online workshop. Walking the talk, most of our participants became co-researchers and co-authors of this paper.

Firstly, members of the research team read through all the transcripts from the recorded conversations and noted down their thoughts and reflections on co-production practices within each CLAHRC and Fuse and barriers and facilitators in using these practices. Research team members did this first separately and, secondly, compared notes and reflected collectively in a joint interpretation meeting on 18 February 2021. This resulted in the identification of six tensions that were apparent when applied health research was co-produced within the CLAHRCs (see the “Results” section). Thirdly, the collective reflections and analysis from the research team were shared with the interviewed CLAHRC theme leads in an online workshop on 12 October2021 to facilitate collective sensemaking.

In preparation for the workshop, senior leaders were tasked with completing a resource pack (Additional file 2: Appendix 2) that summarised the six tensions identified by the research team in their joint analysis meeting. They were asked to comment and make suggestions for each tension and subsequently rearrange the tension cards according to how important and/or relevant they are to the present Applied Research Collaborations (ARCs) using an inner (most important and/or relevant) and outer circle (least important and/or relevant).

This three-step approach to the analysis of the conversation data facilitated the recalling and organisation of the research team’s memories of the conversations and supported self-introspection to analyse these memories. To select memories, senior leaders were asked during the workshop to reflect and add to each tension through a group discussion, in which we were also checking for shared meaning of the tensions. At the beginning of the workshop, senior leaders were asked to nominate their most important/relevant tensions in a poll, which formed the basis for the discussion. Based on this discussion, an additional tension was identified (motivation vs lack of skills) and added to the previously identified tensions, whilst three other tensions (4. research vs non-research activities; 5. traditional academic ways of working and publishing vs new way of generating and disseminating evidence; 6. strategic leadership vs capacity on the ground) were merged into one new tension to represent the overarching tension of individual motivations versus structural issues, bringing the final number of tensions to four.

## Results

The findings highlight a variety of practices across and between CLAHRCs, with the context in which co-production occurs, and the values, expectations and motivations that collaborative partners applied within their different contexts, determining the nature of the co-production processes and outcomes. The CLAHRCs were based on a model of co-production that was evidence based [21]. However, each CLAHRC was developed in a different context responding to unique local needs, resulting in diverse co-production practices. We highlight these different practices through the lens of four tensions that represent the main challenges that the five collaborative research partnerships had to solve differently to develop their co-production practices. We present these tensions as a spectrum along which senior leaders can move when thinking through their approach to co-production. We identified the following four tensions in doing co-production and below we will discuss each tension in more detail:

1. Idealistic, tokenistic vs realistic narratives
2. Power differences and (lack of) reciprocity
3. Excluding vs including language and communication
4. Individual motivation vs structural issues

### Idealistic, tokenistic vs realistic narratives

Senior leaders reflected on how some co-produced applied research can be tokenistic with passive collaboration (only pulling in knowledge when you need it) and less emphasis on empowerment, equality and inclusion; yet at the same time argued that ‘gold standard’ co-production may not be achievable (and may put people off trying).

From their experience, senior leaders highlighted that there is no one size fits all when it comes to co-production. Different projects require different methods and therefore the definition of co-production needs to be fluid to allow for this.

“One of the things we’ve got to is that co-production isn’t one thing and shouldn’t be one thing. It’s a bit like the elephant. It looks different, depending on which direction you approach it from.”

Setting a “gold-standard” method/definition for co-production was felt to discourage researchers from trying to work in co-production and, therefore, a balance is needed between aspirations for co-production of research and what is realistically achievable, given different contexts and limited resources. Getting this balance wrong, e.g. not making choices about what is feasible and being unclear about the realities of what is achievable, risks tokenism.

Tokenism came up several times in the conversations and was linked by senior leaders to both a lack of consistent terminology in the use of co-production and a lack of funding for co-produced research, which we discuss as two sub-themes with this tension below.

#### Lack of clarity on the meaning of co-production

Senior leaders reflected on a lack of general consensus about what is and what is not considered to be effective co-production and how this can lead to confusion and ambiguity. In practices across the CLAHRCs, the terminology around co-production varied considerably. Terms used included: co-design, translational research, co-production, co-creation, knowledge mobilisation and patient and public involvement (PPI). Although, the senior leaders felt there was some overlap in the meanings implied by these terms, many considered them different forms of involvement and their loose definitions lead to confusion. (For a more detailed discussion of these different terms, see our scoping paper; ref).

In addition, senior leaders suggested that many health professionals are doing co-production research under a different name or by using differently terminology. This makes it difficult to recognise how many projects are actually working in this way.

“I went back to thinking like a nurse and thinking about the knowledge-practice gap. And that’s what translation is and then I was looking at integrated knowledge translation and co-production, I thought, well, this is what we’ve been doing, but we were calling it shared decision-making and you’re calling it translated knowledge into action.”

According to collaborative research partnership leaders, this lack of defined terminology can open the door for tokenistic involvement: “Tokenism takes advantage of the elasticity of definition or specificity of co-production.”

PPI was particularly highlighted by the senior leaders in terms of its similarity or difference to co-production. They felt that PPI was already well defined [30], but it is not necessarily clear how it differs from co-production, with some people seeing these terms as two ways of describing the same thing: involving external stakeholders in research, either as patients, public members or practitioners and policy makers.

Other senior leaders argued that PPI equated to more passive involvement, with co-production encouraging more active involvement of outside groups through power sharing. Moreover, co-production does not always involve patients or the public: stakeholders from outside academia can come from a variety of fields and (professional) backgrounds.

Senior leaders also distinguished co-production from dissemination of research. Co-production began early and was seen as more than the re-packaging of research findings at the end of the line to be gifted to external stakeholders.

“I think increasingly I’m realizing that levels of understanding about what we mean by co-production are so massively varied… there are people in senior positions in the academic hierarchy who still understand co-production as being about the dissemination of research findings. Once you’ve done it, basically you’ve bundled it up in a neat package and you’ve written some briefing or some such. And that view persists. And that’s a really hard one to shift, [..] unless NIHR starts taking it more seriously and understanding that it happens right the way through the research process from start to finish and beyond, I think it’s really, difficult.”

#### Lack of funding for co-produced research

Tokenism in practicing co-production was further fuelled in the eyes of senior leaders by a lack of funding for meaningful co-produced research. They commented on the increasing requirement of funders to work in co-production with insufficient resources being made available by funders to commit the time and effort needed to drive good co-production practices. There was a feeling amongst the senior leaders that a technocratic view of co-production (breaking it down into distinct and manageable parts with separate resources) leads to tokenism, which de-values co-production as a concept. They argued that stakeholders involved in a tokenistic way would be less likely to engage with co-production of research in the future, as they felt unheard or under-valued when sharing their experiences.

“.. tokenism talks directly to the fact that if you don’t have money to do it properly, you don’t do it”.

Other senior leaders commented that some funders do not fully understand the activities and engagement that co-production actually requires and at what stage.

“But then also I don’t think that the way that NIHR function and the kind of things they ask for in bids for funding really…they don’t really understand the nature of the engagement that is necessary.”

“People do it as cheaply as possible and as quickly as possible and that will you get what you pay for. So, I think there really needs to be a recognition, if they want really good co-production and patient public involvement…That has to be funded.”

The way research is delivered in terms of funding applications and ethical approval for projects means it is hard to engage stakeholders in the earlier design phases of research. This then makes it harder for stakeholders or members of the public to influence the direction of the research when a plan is already approved and in place.

According to senior leaders, an important condition for co-producing research is creating meaningful relationships with stakeholders to allow trusting and equal partnerships. Creating these contacts and relationships however is not considered in project funding or planning.

“You can’t build relationships with people if nobody’s paying your salary at the point where you need to be doing it, for example.”

Although many funding bodies and research teams say they support co-production, as soon as funding becomes tight, it was felt that protected time for co-production is one of the first things to suffer.

The senior leaders explained how the CLAHRCs were able to make a difference to the funding available for co-production of research by including co-production as a core principle in their business model with dedicated funding.

“In the Autumn of 2008 we held a co-design workshop with all our South Yorkshire stakeholders and academics, the purpose of which was to establish core principles and ways of working. At this point, we developed and approved our core principles, one of which was co-production. We developed mechanisms to achieve and enable co-production and then implemented this core principle across the lifetime of the South Yorkshire CLAHRC.”

An example of funding mechanism in the South Yorkshire CLAHRC was the Getting Research Into Practice (GRiP; see case study in Supplementary files) programme:

“The GRIP programme was a series of co-design projects the purpose of which was to get research into practise. This has gained national recognition in the field of co design and co-production.”

Although the CLAHRCs, were able to tackle the funding issue around co-production to reduce tokenism, the issue of lack of clarity about the meaning of co-production remained. Therefore, senior leaders called for more transparency about what researchers mean by co-production and the extent to which stakeholders outside academia were included throughout the research process.

### Power differences and (lack of) reciprocity

Academics often see themselves as ‘experts’ and need to recognise ‘experts by experience’ as equally powerful; everyone involved should gain from co-productive evidence generation. Senior leaders identified the need to challenge traditional academic research approaches and to be flexible and creative in co-production, which will be explored below as two sub-themes within this tension.

They mentioned repeatedly the tension of power sharing, subscribing to the ideal of equal power relations as a prerequisite for co-production. Power sharing is essential for building good relationships and recognises the value that practitioners, policy makers and members of the public can bring in terms of knowledge, skills and experience in co-producing research. However, achieving power sharing proved difficult in practice.

The senior leaders described different examples of groups outside academia who participated in their CLAHRCs. These included both individuals, small groups and larger organisations. Examples included healthcare professionals, policy makers, patients, funders, commissioners, local community groups, technical experts, public committee members, services users and private sector groups. Many of the researchers talked about the ways in which these stakeholders had participated in different research projects, such as facilitated workshops, knowledge exchange events, peer researchers (e.g. stakeholders as interviewers) and stakeholders working in an advisory group to help steer the direction of research.

One example discussed involved the use of Lego serious play to deliver a shared model of co-production.

“What was particularly novel in the Yorkshire and Humber CLAHRC was the development of a concept known as creative practise, led by Dan Wolstenholme and Joe Langley. It was a programme of work that used co design to co-produce knowledge mobilisation tools”.

Another team recommended setting ground rules at the start of the session to ensure everyone was on the same page and felt comfortable to share their ideas and experiences.

#### Challenging traditional research approaches

Much of the conversations between the senior leaders and the research team focused on the challenges of doing co-production in the landscape of clinical academic research. Co-production challenges traditional (e.g. positivist) research approaches and requires a change in how researchers view their roles as academics.

“But that means giving up a bit of power and you know we’re good at beaming in as the expert because that makes us feel good. We’re not very good at beaming in, and it takes a brave person to say, I haven’t got all the answers, tell me what you think might work. And it completely flies in the face of everything that people think that their role or they’ve been taught their role as an academic is all about.”

For co-production to be successful and produce outputs which are valuable to the involved stakeholders, senior leaders argued that academics need to be willing to compromise on things such as research direction and project design. They acknowledged that this change in academics’ usual way of working would be new ground for many researchers and can be both unfamiliar and uncomfortable, to the point that some academics would feel that their academic integrity was being compromised.

“What I wanted was open mindedness and flexibility, to come to a sort of mutually agreed project spec and scope on the basis that it would be more likely to be achieved. But of course, the mutual agreement often meant, as we’ve looked at it - the kind of compromise and those kinds of issues: academics felt their integrity was being compromised.”

One of the key requirements for working in an equal, power-balanced way with external stakeholders highlighted by the senior leaders was the ability for academics to be flexible in the research process and choice of methods. Over time, the priorities and direction of stakeholder (and academic) organisations may change. This can be challenging to address when projects have already been outlined and funded, but flexibility to adapt to the needs of stakeholders was deemed crucial. This flexibility was not seen as available in the current research and funding system.

“There’s this whole sort of set pathway where you plan ahead for the next five years, what you will be doing that doesn’t leave any space to have these early conversations where you say, well, actually scrap that what we really should be doing is this. What is it that you think we should be doing? You know, what do you think is important?”

The senior leaders did not refer to a flip of power, whereby researchers would completely defer to their practice partners but suggested instead more of an active negotiating process in which health professionals and policy makers have equal power to make decisions about the research. This requires an additional set of skills from those typically associated with academic researchers, including humility.

#### Co-production as a creative endeavour

Co-production was described as ‘a creative endeavour’ which does not sit very well within rigid pre-determined research structures and processes:

“There is something quite rigid in the way that some forms of research, people are trained and taught. I mean the idea that even after participant number two you know something is not going to work. But because you’ve got a sample of however many participants in your trial, you have to pursue it right to the bitter end. That kind of inflexibility is…I might be exaggerating, but that kind of inflexibility is something which is a whole paradigm of research. And it’s deeply engrained, it’s cultural. And co-production is creative, emergent, responsive, all of those opposite things”.

Whilst more rapid research designs or rules for stopping in traditional clinical trials reduce some of rigidity in research, the perception of the senior leaders was that more flexibility is required in co-producing research.

One researcher in the North East discussed an example where they were embedded in a community and asked to develop responses to tackle childhood obesity. Early conversations with community members indicated that they were more concerned with poverty, inequality and the early roll out of Universal Credit, leading to a follow up study being commissioned on the impact of Universal Credit:

“And, you know, the Universal Credit study is a brilliant example. And it started out with you know, a project which was supposed to be about childhood obesity, because that was an issue. But then the local community said, no, we*’*re less concerned about childhood obesity and more concerned about Universal Credit actually, because that affects our very survival.”

This example, points to another potentially important trait for co-production research: starting small can develop trusting relations for larger projects, with organic development of research projects being much more conducive to co-production processes involving wider groups of stakeholders.

“there was quite an impact from, and I, sometimes I forget about the you know, that, again, it started from a small scale, small-funded project, and then ended up with (researcher) talking to it, to the select committee and, you know, and, and that then resulted in some supermarkets restricting sales to energy drinks to under eighteens or under sixteens in some cases.”

Working flexibly with stakeholders during the research process also requires from academics an understanding and appreciation about what stakeholders expect or want from the co-production process. Stakeholder involvement was viewed as a two-way street. Senior leaders emphasised that, although we may have an ideal as academics of how we want from stakeholders’ input, we need to be able to adjust for how much or little they want to get involved. Whether that is down to the time and resources they can feasibly spare or how much they are wanting to engage and participate, we need to work flexibly and have early conversations about expectations around involvements and outputs. For instance, for many stakeholders, getting papers published was not a reason to get engaged with research: “Publications are not sufficient for many participants. The difference work makes has to be real to them.” These power difference also extended to tensions between academic researchers within the CLAHRCs (see case study 2 in the Supplementary files).

### Excluding vs including language and communication

The use of ‘research’ jargon and the communication style of researchers can exclude partners involved in co-production such as service users, managers, or practitioners. Senior leaders highlighted the importance of language and communication in co-production and the need for more training in co-production craft (the skills in the practices and activities of co-production) to, which will be discussed below as two sub-themes within this tension.

#### Language and communication

The senior leaders emphasised that language and communication skills were very important in co-produced work, both to help build relationships and to make data and research ideas accessible to all involved stakeholders.

“Different people learn, communicate and express themselves in different ways. Using only forms that are common to researchers, excludes some.”

They urged academics to try a variety of different engagement techniques and communication styles to get the best out of co-production with different stakeholder groups. However, these types of skills aren’t necessarily held by all academics.

“You do have to use lots of different methods in order to get the most bang for your buck out of your research. And actually taking some of that time up front to use better methods to engage means you get better engagement.”

Another skill suggested by the senior leaders for co-production of research was the ability to find and engage with the right people within stakeholder organisations. How to identify key people and how to connect with them in a meaningful way was perceived by them as an ongoing challenge, particularly in larger organisations, such as local government or NHS Trusts.

“The partners that we had most difficulty engaging tended to be the larger acute organisations because you can’t engage with a whole organisation and it’s finding out who the key people are… So, some of the problem was identifying the right people to talk to and you could be passed from pillar to post.”

Senior leaders suggested that academics do not always need to have the necessary design skills themselves but can broker links with other colleagues within their institution or networks or in other departments within their university, such as design students. These colleagues and students can add creativity and bring a fresh prospective to the research.

“So, I think one of the big things that we pushed a lot was look to other parts of your university, look to the design departments, for people who can come up with ideas or visualize things that your team can’t.”

Working in co-production was perceived by the senior leaders as a unique craft requiring different skills that need constant attention through the research process. They defined this craft as skills in the practices and activities of co-production that were developed through experience (to develop the art), combined with knowledge (based on the science) of coproduction. They advised building in regular moments for reflection and reporting in team meetings on how the research team is practicing and achieving co-production. The floor should be open for teams to consider how they are involving their stakeholders and whether anything else can be done to facilitate further meaningful engagement/involvement.

“Co-production doesn’t just happen. It’s not just, it’s not just bringing people together in a room. It was a very, very conscious attention to a whole range of factors that allows good co-production to happen.”

To support this reflective process, one leader suggested that teams appoint co-production champions at all levels of their organisation to promote collective reflection and building capacity and capability in co-production.

“Even if you don’t have a dedicated theme, you need dedicated champions and those champions need to be scattered throughout the organization, different positions at different levels.”

Another way suggested by senior leaders to build this capacity and capability in the research system was by incorporating co-production training into undergraduate, Masters and PhD programmes. Co-production is currently not built into the curriculum of academia. Instead, they advocated for more teaching early in academic careers about different ways of doing research and valuing different ways of knowing. It was felt that good policy influencers require changes to the academic models that produce them. The biggest barriers to co-production were thought to be structural and often located in academic institutions (see tension 4 below). As long as we do not train students in engaging with policy and practice partners, fail to teach and reward them in how to use different types of evidence and do not involve them in collaborative research, we will keep returning to the conclusion that very little research evidence is getting used in practice and policy.

#### Motivation versus skills

The lack of training in co-production is central to the four tensions that senior leaders identified: the tension between an individual’s desire and motivation to work in co-production with external stakeholders on research (which varied within CLAHRCs) and their capability and capacity to do this and deliver it in projects.

“Looking back through our CLAHRC is that I think there were some tensions between motivation to do it, but not having the skills or abilities to deliver. So, some of it was actually more within individuals or projects.”

They outlined co-production skills as a separate skill set that cannot be taught in a two-day training course but needed to be acquired through practice, for example, being flexible, persuasive, planned happenstance, enthusiasm, serendipity, perseverance, patience, negotiation, pragmatism, learning-oriented, empathy and confidence [31]. Practicing co-production was seen as understanding different ways of knowing (cognitive flexibility). Whilst it is important to give people a go at working in co-production, senior leaders felt it was important for them to consider the skills that are needed to work in this way and who they could bring in as part of their research projects to facilitate those skills (e.g. mentorship). Researchers do not need to be experts themselves but could learn on the job from these experts:

“You need to appoint someone to facilitate and lead co-production who is skilled and expert at doing it. And, therefore, there needs to be a process where you enable people to enquire and accumulate those skills perhaps under the supervision and mentoring of people and participating alongside people who are more skilled at doing it. Because that way it shows respect and value to the whole process of co-production itself”.

Involving co-production expertise from the start in research projects, next to other roles such as statisticians and qualitative researchers, was seen as an important mechanism to support and teach other team members in developing their co-production skills and to build co-production capacity within research teams. The senior leaders suggested moving away from a perception of co-production as a soft skill and defining it more as a craft that researchers need to hone and develop over time. Using the right language and communication about co-production includes how these skills are defined and labelled.

### Individual motivations versus structural issues

#### Individual motivation for working in co-production

Despite a lack of clarity around the meanings of co-production, lack of co-production skills and a lack of funding for meaningful co-production, senior leaders generally highlighted positive experiences of working in co-production with stakeholders in the CLAHRCs, both from an instrumental and moral imperative. Instrumentally, the senior leaders linked the impact agenda and negative perceptions of the public about research as incentives for engaging in co-production of research. Applied health care research can sometimes be seen as the nanny state, finger wagging and patient-blaming, but that image can be changed by academics working on issues that matter to the public and that hold value for the stakeholders involved in co-production.

“It can be very rewarding because in terms of the kind of impact agenda for some academics they can see real benefit in the work that they’ve done being used, enabling change in practice, etc.”

Senior leaders highlighted from their experiences how co-production improved the quality and utility of their work. Involving the end-users in the design and development process, participants felt that they were more likely to come up with a product that was fit for purpose and better suited the needs of their target audience.

“Pragmatically if you work with the people who are going to use the stuff that you were trying to make, be that research services, products, whatever, they were more like to use them in the long term. Pragmatic logic that co-designing services and products means people more likely to use them. So, you got better stuff. You got better things out the other end.”

This requires a critical look at the distinction between research users and producers. Academics are not the only ones producing research, and patients and the public are not always end users [32]. Within research partnerships, stakeholder involvement allowed for better knowledge to be created and shared by making use of knowledge from lived experience.

Morally, the senior leaders felt that people should be included in research and projects that impact them. They referred to similar imperatives in other disciplines, ranging from commercial groups using consumer testing and feedback, to healthcare authorities emphasising a patient-centred, shared decision-making approach to patient care to highlight stakeholder involvement as business-as-usual in health and social care sectors. Therefore, including stakeholders in research was seen as the right thing to do.

“On one level, we absolutely believed that co-production, as in working together with people and patients, was the right way to go about doing things.”

#### Structural barriers

However, the ability and capability to work in co-production in the CLAHRCS was to an extent dependant on wider structures and system incentives, which often hampered opportunities for academics to engage in meaningful co-production with external stakeholders. Co-producing evidence means researchers enabling people’s involvement, partnership engagement and facilitation; academic institutions tend not to recognise or reward these non-research activities. Senior leaders complained about academic institutions not facilitating or valuing co-production practices. The outputs of co-produced projects are not necessarily traditional high-impact papers, and many senior academics see co-production as a lower rung in the research evidence hierarchy, which is not conducive for academic promotion.

“I think also the structures in which academia works, doesn’t value, the outputs of co-production because they aren’t papers.”

“What I find sad is that the people who genuinely had that much more partnership engaged approach are not the ones who are seen as great academics and I think that’s a shame, but I think that’s a problem with the academic system.”

Moreover, traditional academic, positivist ways of producing evidence value objectivity and separation of researchers and participants, whereas working in a co-productive way involves generating experiential knowledge, sharing of roles and more dynamic and equitable relationships across the research cycle (see case study CLAHRC South Yorkshire: utilising different skills sets).

Some senior leaders within CLAHRC played a critical role in envisioning co-production within their research structures, although the capacity to enact and use co-production in projects varied. In the discussion of our first tension (on idealistic, tokenistic vs realistic narratives), we saw an example of how leadership in a CLAHRC ensured that co-production principles were encouraged as a way of working within the structure from the start. However, encouraging all members of the CLAHRC to apply these principles proved an ongoing challenge.

“When I then put together the Yorkshire and Humber (YH) application we carried these core principles into the YH CLAHRC. However, this was a more difficult challenge, as the geography was huge and the concept wasn’t as well understood amongst some academics. Over time, running workshops and marketing materials such as our brochures and ‘Bite’ we did achieve co-production but perhaps not in all themes”.

Some senior members of academic institutions who make decisions about funding, impact case studies and publication fees, do not value co-production as they have not been exposed to it in their career or do not appreciate its role as a form of valid research.

“In a way the system has rewarded people who’ve got to those very senior decision-making positions, and a lot of them have got to where they are without needing or wanting to work in a co-production way. And so, in a way, what’s the incentive for people to change and do more of that because you know that they’ve got where they are, and they’ve done very nicely out of it.”

It was recognised that although junior members of organisations usually have more time and energy to engage stakeholders and public contributors in research projects, they do not necessarily have the power and influence in the organisation to make co-production a priority.

As Pearce [33] points out in several studies [15, 34, 35], much of the work of PPI and co-production is carried out by those on the ‘lower’ end of the academic hierarchy, such as junior researchers who are likely to have short-term contracts. The gendered and racialised aspects of co-production have also been highlighted [15, 36], with women and ethnic minorities tending to carry out the labour of research, whether as academic, peer researcher or patient and public member, but who in terms of secure employment and research funding may hold little power.

Conversely, the people at the top of the organisation with the influence, often do not have the time and resources to commit to these co-produced projects.

“I would be worried, if people tell you they’ve got lots of time to engage with you they’re probably not the key people in the system because the key people in the system are very overwhelmed.”

However, senior leaders were keen to stress that co-production is a human resource process that needs people. Junior researchers need to be encouraged to go into co-production processes, just as they have permission to develop their partnerships for research applications. The role of senior leaders was seen as enabling this. Complexity of organisations and research infrastructures, such as CLAHRCs, can make this a challenge with leaderships spread across different levels and therefore potential blockages in junior researchers receiving permission for co-production.

“There’s the very strategic leadership of the CLAHRC and then there are leaders within the themes as well. And both can be enabling, or they both can be blocking. [..] Within our CLAHRC we have principles, and co-production was one of them, and we asked people to reflect on what that meant for them. But it could be that a theme lead didn’t really understand or know the difference between co-production… there would be differences in those concepts. And they could block it, or they could enable it through the use of a resource”.

Therefore, senior leaders suggested a need for coordination between multiple levels of leadership to enable co-production, particularly around resource allocation for co-production.

“Some discontent, shall we say, [within our CLAHRC] about resources being allocated to non-research. Resources were still allocated to non-research but there was a lot of discussion and negotiation at senior level. And explanation as to why we have to do it”.

## Discussion

We identified four tensions in doing co-production that the five collaborative research partnerships had to solve differently to develop their co-production practices: (1) idealistic, tokenistic vs realistic narratives; (2) power differences and (lack of) reciprocity; (3) excluding vs including language and communication; and (4) individual motivation vs structural issues. These tensions highlight different dilemmas that the collaborative research partnerships faced in developing their co-production practices, requiring each partnership to develop a response to these tensions, taking into account local context, needs and existing opportunities and partnerships. Therefore, each partnership responded differently, resulting in different co-production practices. In other words, these tensions were productive. Below we highlight two take-away messages that we identified from our joint reflections with senior leaders of these collaborations.

### Key take-away messages

#### No gold standard: variety of co-production approaches for developing context-tailored solutions

Our first point of reflection is that these variations should not be reduced to one gold standard for co-production but should be celebrated and understood in the context in which they were developed. This will help other research infrastructures, such as the NIHR ARCs, HDRCs and social care research networks, to reflect on how to practice co-production in their organisational structures and context. Reimagining challenges as tensions encourages academics and health professionals to articulate their positions on co-production more carefully and also emphasises that one size does not fit all in co-production.

Power differences underlie many of the other tensions; facilitating power sharing in co-production activities is, in our experience, crucial for finding solutions to the challenges that other tensions pose. This is also acknowledged in the literature by Williams et al. [37], who point to the dark shadows cast on co-production, caused by underlying structural issues of power (particularly in academic institutions).

In our study, we have shown that power sharing requires new roles and approaches from academics to respond with flexibility to stakeholders’ needs and changing engagement across contexts, ensuring inclusive language and communication. Senior leaders need to empower junior researchers to get involved in co-production by providing them with sufficient resources and co-production skills, giving them enough space to experiment (and permission to fail) by changing the structures in which they operate.

Perhaps this is the real aim of co-production in research: not to co-produce new knowledge but to reconfigure the structures in which this knowledge is enacted. Miller and Wyborn [38] argued that the purpose of co-production is to create new forms of governance that produce the required knowledge and at the same time the social dynamics to act on this knowledge. In line with their work, we propose to frame co-production as a creative space to experiment with and develop new governance structures.

#### Addressing structural barriers: distributed leadership

In many of the tensions, the starting position will be determined by the vision and values of the collaboration leaders. Bringing together a range of organisations and people in a new complex collaboration requires the formative role of a (individual) leader to shape the architecture of the collaboration, with the vision and beliefs of this leader influencing the approach to co-production. However, as collaborations such as the CLAHRCs evolved over time, new models of leadership (e.g. distributed leadership [39]) developed that facilitated more power sharing across the collaboration (Kislov R, Harvey G, Bresnen M: Supporting the transition from individualistic to collective leadership: a longitudinal study of a university-healthcare partnership, forthcoming) and strengthen structural conditions for co-production. These new models of leadership are more focused on engaging stakeholders and taking account of local contextual factors, and they require the individual leader to relinquish some of their control to other senior leaders in the collaboration, creating more uncertainty and ambiguity that they need to feel comfortable to manage (Kislov R, Harvey G, Bresnen M: Supporting the transition from individualistic to collective leadership: a longitudinal study of a university-healthcare partnership, forthcoming).

Not every leader is keen to share power and we identified in our conversations with CLAHRC leaders’ differences in the extent to which senior leaders are willing to relinquish their control to others. For example, the CLAHRC South Yorkshire/Yorkshire and Humber developed a system of distributed leadership. Resources were allocated to the themes and theme leads then had the power to use these how they wished. The balance and use of resources really reflected the belief in co-production within the theme leadership. This was visible in budget spreadsheets on how resources were spent, with research often being only one component of the budget with a greater mix of funding being allocated to work in co-production and spending time on priority setting with external partners. However, some theme leads just used funds to do traditional research, illustrating that distributed leadership gives freedom to use resource agreed at the senior level, but that this played out differently at theme level.

### Practical implications

We recommend that the four tensions should be acknowledged and worked through by senior leaders in collaborative research partnerships as constructive dilemmas to enable effective co-production. By thinking about their responses to each challenge, senior leaders will be better able to define, resource and implement co-production practices in their work and structures. Rather than seeing these tensions as barriers, we suggest re-imagining them as a creative process that will lead to potential solutions. To support this creative process, we made suggestions for responding to each challenge and present illustrative case studies in supplementary files that illustrate how different CLAHRCs have addressed these tensions.

Our study demonstrates that these tensions were productive in helping collaborative research partnerships to tailor co-production practices to their local needs and existing opportunities. As a result, practices varied across partnerships, which we argue should not be reduced to one gold standard for co-production but should be celebrated. The links between the tensions informed solutions in each context, with strategic leadership identified as an important starting point; however, this role needs to be developed into more distributed forms of leadership over time to facilitate co-production practices between partners. Facilitating structures for co-production enabled power sharing through capacity and capability building, which resulted in more inclusive language and communication, and a virtuous circle resulting in more realistic practices of co-production in research.

#### Creating a realistic narrative around co-production

In this sense, the first tension is not really a challenge but an ambition: how to create a realistic narrative around co-production within a research infrastructure or organisation that is not unachievably idealistic and does not merely present a tokenistic effort? To support this ambition, the other tensions need to be resolved by making a choice about where to start with developing your co-production practices. Navigating these tensions is a craft in itself which can only be developed through practise. However, asking yourself a few questions as a team of leaders before you embark on your co-production activities together will help you work out your collective responses to the three other tensions.

**Table:** 

*Questions for responding to the four tensions of co-production in collaborative research partnerships:* • What is our vision for co-production? How do we define it and embed this in our organisation’s strategies and structures? • What language and communication will be helpful to share this vision within and outside the collaboration? • How much power are we willing to share with other senior leaders in the collaboration? And how will we manage uncertainty and ambiguity resulting from power sharing? • How much capacity do we have in my organisation to support co-production? And what can we do to increase capacity/capability of existing staff? • What resources will we need to for this and how do we distribute them across the collaboration? • How can we reflect on progress in realising this ambition at regular intervals with external partners?	

#### Facilitating power sharing, inclusive language and co-production skills

Facilitating power sharing in co-production activities is, in our experience, crucial for finding solutions to the challenges that other tensions pose. We suspect that a truly egalitarian sharing of power within these collaborations will be hard to achieve; however, more distributed and collaborative forms of leadership, facilitate co-production [22]. Distributed leadership can be facilitated by more inclusive processes and governance structures within collaborative research partnerships, including, for example, rotation of chairing responsibilities within the team, attempts to open the agenda-setting process to all team members, and efforts to make dialogue a more prominent feature of the team meetings.

Embedding co-production practices in collaborative research partnerships can be further supported by organising regular reflections with both internal and external stakeholders. For example, by organising action learning sets or developing communities of practice to reflect and report on how they are achieving co-production. These reflections encourage collaborative problem solving, whilst celebrating success and learning from failure, creating more inclusive language and communication.

Finally, practising co-production requires a very different skill set of academic researchers in terms of communication, relationship building and power-sharing, which is not currently taught in academic curriculums, and take time to master. The insights from senior leaders of collaborative research partnerships shared in this paper demonstrate that this skill set is more of a craft that needs to be honed and nurtured over time.

We argue for the need to educate all researchers about strategies for making their research more relevant, applicable and impactful. Co-production approaches could be an important element of this. At the same time, we acknowledge that deep engagement with co-production and successfully addressing its tensions would require considerable experience and expertise. This could be achieved by some researchers specialising in co-production methods—but also by developing the cadre of knowledge brokers and hybrid roles (embedded researchers, practitioner fellows) who straddle the communities of ‘knowledge production’ and ‘knowledge application’ (Kislov R, Harvey G, Bresnen M: Supporting the transition from individualistic to collective leadership: a longitudinal study of a university-healthcare partnership, forthcoming).

### Strengths and weaknesses

The auto-ethnographic approach taken in this study allowed for in-depth reflections with senior leaders on the tensions they faced in developing co-production practices in their collaborative research partnerships and a process of collaborative sense making with research teams. This way of working is illustrative of the topic of this study: not only did we co-produce the study; we also co-produced this paper with the research participants. However, the findings are based on the reflections of the research team and a limited number of senior leaders from collaborative research partnerships, which may limit generalisability to other settings.

Whilst we feel that these tensions adequately represent the most significant issues we experienced in co-production in the five partnerships, we are mindful that these partnerships are set within an English context and therefore different tensions might apply in other countries with different governance and health systems. However, the literature suggests the ubiquity of these challenges [40] and, whilst there may be much to learn from other jurisdictions where the health systems and governance arrangements may differ, some of the underlying tensions that determine co-production will be similar [41].

Our focus in this study is on the experiences and perceptions of senior leaders of the four tensions and how they tried to solve these tensions. It is unknown to what degree these overarching narratives were shared within individual collaborative research partnerships across different members and partners. However, the findings of our study suggest the importance of formal leaders’ visions in shaping the partnerships’ architecture and vision and, therefore, their perceptions and experiences are important to focus on (Kislov R, Harvey G, Bresnen M: Supporting the transition from individualistic to collective leadership: a longitudinal study of a university-healthcare partnership, forthcoming).

## Conclusion

Despite a growing enthusiasm for co-production in healthcare services and research, there is noticeable gap in the current literature on ‘how-to do co-production’ in large partnership structures. In this auto-ethnographic study with senior leaders from five successful collaborative research partnerships in the UK, we reflected on co-production practices between academics, health professionals, policy makers and third sector organisations to inform practical guidance on co-production for new partnerships, such as the NIHR Applied Research Collaborations (ARCs).

## Supplementary Information

**Additional file 1.** Completed Standard for Reporting Qualitative Research (SRQR).

**Additional file 2: Appendix 1.** Story line topic list for interviews. **Appendix 2.** Resource pack for interactive workshop.

**Additional file 3:** **Supplementary Files.** Case studies of the four tensions.

## Data Availability

The datasets used and/or analysed during the current study are available from the corresponding author on reasonable request.

## Abbreviations

ARCs: Applied Research Collaborations
CLAHRC: Collaborations for Leadership in Applied Health Research
GRiP: Getting Research Into Practice
NIHR: National Institute of Health and Care Research
PPI: Patient and public involvement
UKCRC: United Kingdom Clinical Research Centres
UNICEF: United Nations Children’s Fund
WHO: World Health Organisation
