# Correction: Leading co-production in five UK collaborative research partnerships (2008–2018): responses to four tensions from senior leaders using auto-ethnography

**DOI:** 10.1186/s43058-023-00401-x

**Published:** 2023-02-24

**Authors:** Peter van der Graaf, Roman Kislov, Helen Smith, Joe Langley, Natalie Hamer, Mandy Cheetham, Daniel Wolstenholme, Jo Cooke, Sue Mawson

**Affiliations:** 1 Northumbria University, Newcastle upon Tyne, UK; 2 Manchester Metropolitan University, Manchester, UK; 3 University of Manchester, Manchester, UK; 4 National Institute for Health and Care Research, Liverpool, UK; 5 Sheffield Hallam University, Sheffield, UK; 6 Newcastle University, Newcastle upon Tyne, UK; 7 Royal College of Obstetricians and Gynaecologists, London, UK; 8 Sheffield University, Sheffield, UK

**Correction: Implement Sci Commun 4, 12 (2023)**

**https://doi.org/10.1186/s43058-022-00385-0**

Following publication of the original article [1], an error was identified in the Funding section.

The updated Funding statement is given below, and the changes have been highlighted in **bold typeface**.

**Funding**

Funding from the National Institute for Health and Care Research (NIHR) School for Public Health Research (SPHR) is gratefully acknowledged for resourcing NH to support the data analysis and write up as part of her Summer Internship. RK is partially funded by the (NIHR) Applied Research Collaboration Greater Manchester (ARC-GM). PF and MC are members of the NIHR Applied Research Collaboration North East and North Cumbria (NIHR200173). **HS is funded by the (NIHR) Yorkshire and Humber ARC (NIHR 200166)**. The views expressed in this publication are those of the author(s) and not necessarily those of the National Institute for Health and Care Research or the Department of Health and Social Care.

The original article [1] has been corrected.
