# Supplementary material for: Leading co-production in five UK collaborative research partnerships (2008–2018): responses to four tensions from senior leaders using auto-ethnography
Source: Implement Sci Commun. 2023 Jan 27;4:12. doi: 10.1186/s43058-022-00385-0 (PMC9883908; doi:10.1186/s43058-022-00385-0)
Supplement: Supplementary file 1 — Additional file 1. Completed Standard for Reporting Qualitative Research (SRQR). [file 43058_2022_385_MOESM1_ESM.doc]

Additional file 1.

**Completed Standard for Reporting Qualitative Research (SRQR)**

O’Brien B.C., Harris, I.B., Beckman, T.J., Reed, D.A., & Cook, D.A. (2014). Standards for reporting qualitative research: a synthesis of recommendations. *Academic Medicine, 89(9)*, 1245-1251.

| **No. Topic** | **Page** |
| --- | --- |
| **Title and abstract** |  |
| S1 Title | 1 |
| S2 Abstract | 2 |
| **Introduction** |  |
| S3 Problem formulation | 1, paragraphs 1-4 |
| S4 Purpose or research question | 1, paragraphs 5 |
| **Methods** |  |
| S5 Qualitative approach and research paradigm | 4, paragraphs 1,5  5, paragraphs 1-4 |
| S6 Researcher characteristics and reflexivity | 4, paragraph 4 |
| S7 Context | 4, paragraph 4 |
| S8 Sampling strategy | 4, paragraph 4 |
| S9 Ethical issues pertaining to human subjects | 22, Ethics approval and consent to participate |
| S10 Data collection methods | 4, paragraph 4 |
| S11 Data collection instruments and technologies | Appendices 1-2 |
| S12 Units of study | 4, paragraphs 2-3 |
| S13 Data processing | 4, paragraph 5  5, paragraphs 1-4 |
| S14 Data analysis | 4, paragraph 5  5, paragraphs 1-4 |
| S15 Techniques to enhance trustworthiness | 5, paragraphs 2-4 |
| **Results/Findings** |  |
| S16 Synthesis and interpretation | 5-15 |
| S17 Links to empirical data | 5-15, various quotes in text from interviews and workshops transcripts |
| **Discussion** |  |
| S18 Integration with prior work, implications, transferability, and contribution(s) to the field | 15-18 |
| S19 Limitations | 18, paragraphs 4-5  19, paragraph 1 |
| **Other** |  |
| S20 Conflicts of interest | 22, Competing interests |
| S21 Funding | 22, Funding |
