# Supplementary material for: Leading co-production in five UK collaborative research partnerships (2008–2018): responses to four tensions from senior leaders using auto-ethnography
Source: Implement Sci Commun. 2023 Jan 27;4:12. doi: 10.1186/s43058-022-00385-0 (PMC9883908; doi:10.1186/s43058-022-00385-0)
Supplement: Supplementary file 3 — Additional file 3: Supplementary Files. Case studies of the four tensions. [file 43058_2022_385_MOESM3_ESM.docx]

**Supplementary Files. Case studies of the four tensions.**

1) Idealistic, tokenistic vs realistic narratives

**Case study CLAHRC SY/YH:** **Getting Research Into Practice (GRiP)**

CLAHRC partners in Yorkshire and Humber could apply for funding to undertake a Getting Research into Practice (GRiP) project by submitting a written proposal of maximum four pages. The funding supported the release of staff from Sheffield Teaching Hospitals NHS FT to conduct a research project on an aspect of care provided to patients that could be improved through implementing evidence into practice. GRIP projects were supported by members of the Translating Knowledge into Action theme of NIHR CLAHRC Yorkshire and Humber, with up to three projects funded per year and a maximum of £12,000 for each project. A case book was produced at the end of the CLAHRC to highlight various GRIP projects and the impact they had on practice.

One of the GRIPs projects explored ways to promote exercise in stroke survivors living in Sheffield, using co-production workshops to better understand support from the service users’ point of view. The multi-disciplinary project team, composed of health professionals and designers, used storytelling and visuals to explore myths around exercise after stroke, and to identify key barriers and enablers of services provided in Sheffield. They also co-facilitated a series of five workshops to develop design briefs for creating an ideal service, with support from product design course students at Sheffield Hallam University, who were paired with stroke survivors and healthcare professionals.

This resulted in briefs for a communications campaign to counter myths and promote physical activity, a staff training package to help the consistent delivery of information regarding exercise after a stroke, and the creation of a stroke survivor’s ‘passport’, giving them access to relevant and customised information and keeping their medical information in one place. Briefs were shared with teams across the world to seek funding for developing a prototype to test in stroke wards. For more information about GRiP, please see: <https://clahrcyh.wordpress.com/2016/03/03/getting-research-into-practice-grip-2016-sheffieldhosp-shcfundraising/> .

2) Power differences and (lack of) reciprocity

**Case study CLAHRC South Yorkshire/Yorkshire and Humber: Power differences between academics**

In the CLAHRC South Yorkshire a Knowledge Mobilisation theme was led by a senior academic, while another theme was led by a trialist medic. Both were senior professors in their field; very much driven and fashioned by their considerable experience: one through positivist and experiment(induction); the other driven by theory application (deduction). They respected each other (based on their perceived places in respective hierarchies) and understood that their ways of knowing were different. However, the introduction of collaborative co-production and critical theory/ creative design approaches working was a step too far. They just did not get it and did not use the KM theme’s expertise, as they did not consider it as real research.

There were also power differences within themes. For example, in the CLAHRC’s Obesity theme. We wanted to develop priorities for the theme and do this with services. The priority setting groups included surgeons and public health practitioners. The first group wanted to prioritise tertiary gastric bands and gastric balloons and evaluate this. The second group wanted to look at true prevention, such as sugar tax and changes to the obesogenic environment. Both groups could not compromise and there were real tensions and power issues amongst these stakeholders. In the end we developed collaborative groups working at different stages of prevention to separate the tensions.

3) Excluding vs including language and communication

Language and communication

**Case study CLAHRC Yorkshire and Humber: importance of collaborative agreements between researchers and industry partners**

As an example of research co-production challenges, CLAHRC YH undertook the implementation and evaluation of a project known as the Enhanced Community Palliative Support Service (EnComPaSS), working with the voluntary sector, commissioners and an industry partner. This workforce transformation project in end-of-life care was coproduced from its inception with shared decision-making between knowledge users and researchers, centred around mutual learning and respect (Ariss et al 2021). The success of this project partly lay in the ability of our industry partners to be nimble and responsive to changes in the digital platform requested by clinical teams. However, at times the expectations of both with regards to the feasibility of changes was unreasonable and expectations had to be carefully managed. For example, the need to be clear about foreground and background IP were crucial for new pathway models and novel workforce developments when undertaken in a co-produced way. We learnt the importance of collaborative agreements between our industry partners and all other collaborators in this project. For more information about GRiP, please see: <https://clahrcyh.wordpress.com/2016/03/03/getting-research-into-practice-grip-2016-sheffieldhosp-shcfundraising/> .

Motivation versus skills

**Case study CLAHRC South Yorkshire: utilising different skills sets**

The CLAHRC South Yorkshire developed a three-monthly reporting system that asked theme leads to report on what they were undertaking within their theme based on our principles, with one being co-production. The reporting system worked as a mechanism to share and learn from one another in using co-production skills.

In addition, the CLAHRC South Yorkshire used their Research Capacity Funding to encourage cross fertilisation of ideas and undertake joint projects together. Themes that were experienced in co-production had an opportunity to use these skills with other research themes. This was sometimes successful, leading to more sustained partnerships, but also sometimes did not work. For more information on the CLAHRC South Yorkshire approach to co-production, please see: <https://www.researchgate.net/publication/237151605_NIHR_CLAHRC_for_South_Yorkshire_Internal_Evaluation_Report_November_2011_Executive_Summary> .

**Case study Fuse: AskFuse, a responsive research and evaluation service for public health practitioners and policy makers**

In June 2013, after extensive consultation with local stakeholders and partners, Fuse launched AskFuse: a rapid response and evaluation service to provide decision makers and practitioners with an easy-to-access portal for public health evidence in the North East of England. The service aims to respond to a broad range of research requests from the health, well-being or social care sectors.

The post of AskFuse Research Manager was created to provide a single point of contact for all AskFuse enquiries and to coordinate this service for each client from start to finish. In an initial conversation, the partner’s needs are explored; the nature and timescale of any further work is then agreed over a few meetings (with no obligation or fee), resulting in a research brief for researchers. The costs of any work agreed, and outputs, will be discussed at this stage. The Research Manager then liaises with Fuse senior investigators and staff at the five universities in the North East of England to identify capacity and skills to develop, commission, lead and undertake research projects.

Between June 2013 and January 2022 over 400 enquiries have been supported by the service resulting in more than 150 collaborative research projects and various co-produced knowledge exchange events. However, the knowledge brokering process facilitated by the service has not been without its challenges. For instance, considerable time is often needed to turn enquiries into a format which is ‘researchable’, in part because of unreal expectations. Secondly, local funding for agreed research projects was generally limited, while academic enthusiasm for supporting these projects was sometimes dampened by a lack of institutional incentives to engage in knowledge exchange. Finally, developing AskFuse proved particularly challenging in a time of significant system upheaval in the NHS. This also changed the types of evidence that were valued by enquirers, with more emphasis being put on implementation advice from qualitative or realist designs.

For more information on AskFuse, please see: Van Der Graaf P, Shucksmith J, Rushmer R, Rhodes A, Welford M. Performing collaborative research: a dramaturgical reflection on an institutional knowledge brokering service in the North East of England. Health research policy and systems. 2019;17(1):1-9. Or visit: [www.fuse.ac.uk/askfuse](http://www.fuse.ac.uk/askfuse) .

4) Individual motivation vs structural issues

**Case study Fuse: community-centred approaches to public health/ Impact of Universal Credit in North East England: a qualitative study of claimants and support staff**

The need for the study emerged from embedded research undertaken by an academic researcher within Fuse working with local community groups in Gateshead and drawing on their priorities and experiences. Keen to explore the potential health and social impact of Universal Credit (UC) on residents, Gateshead Council commissioned the study. Local stakeholders were involved in the conduct of the study and in the dissemination of findings.

33 UC claimants with complex needs, disabilities and health conditions and 37 staff from local government, housing, voluntary and community sector organisations were interviewed and took part in focus groups to share their accounts of the UC claims process and the consequences of managing on UC. The findings add considerable detail to emerging evidence of the deleterious effects of UC on vulnerable claimants’ health and wellbeing with evidence suggesting that UC is undermining vulnerable claimants’ mental health, increasing the risk of poverty, hardship, destitution, and suicidality.

The resulting findings were presenting in person to the House of Commons Work and Pensions Select Committee and were cited in a report calling on the Government to provide financial support to people waiting for their first Universal Credit payment.

For more information about this study, please see: Cheetham M, Moffatt S, Addison M, Wiseman A. Impact of Universal Credit in North East England: a qualitative study of claimants and support staff. BMJ open. 2019 Jul 1;9(7):e029611.
